# A class I cytosolic HSP20 of rice enhances heat and salt tolerance in different organisms

**DOI:** 10.1038/s41598-020-58395-8

**Published:** 2020-01-28

**Authors:** Liu-Ming Guo, Jing Li, Jing He, Han Liu, Heng-Mu Zhang

**Affiliations:** 1 Institute of Virology and Biotechnology, Zhejiang Academy of Agricultural Sciences, Hangzhou, 310021 China; 2 College of Chemistry and Life Science, Zhejiang Normal University, Jinhua, 321004 China

**Keywords:** Plant biotechnology, Plant stress responses

## Abstract

Small heat shock proteins (sHSPs) have been thought to function as chaperones, protecting their targets from denaturation and aggregation when organisms are subjected to various biotic and abiotic stresses. We previously reported an sHSP from *Oryza sativa* (OsHSP20) that homodimerizes and forms granules within the cytoplasm but its function was unclear. We now show that OsHSP20 transcripts were significantly up-regulated by heat shock and high salinity but not by drought. A recombinant protein was purified and shown to inhibit the thermal aggregation of the mitochondrial malate dehydrogenase (MDH) enzyme *in vitro*, and this molecular chaperone activity suggested that OsHSP20 might be involved in stress resistance. Heterologous expression of OsHSP20 in *Escherichia coli* or *Pichia pastoris* cells enhanced heat and salt stress tolerance when compared with the control cultures. Transgenic rice plants constitutively overexpressing OsHSP20 and exposed to heat and salt treatments had longer roots and higher germination rates than those of control plants. A series of assays using its truncated mutants showed that its N-terminal arm plus the ACD domain was crucial for its homodimerization, molecular chaperone activity *in vitro*, and stress tolerance *in vivo*. The results supported the viewpoint that OsHSP20 could confer heat and salt tolerance by its molecular chaperone activity in different organisms and also provided a more thorough characterization of HSP20-mediated stress tolerance in *O. sativa*.

## Introduction

Heat shock proteins (HSPs) have been thought to function as molecular chaperones, which are ubiquitous and evolutionarily conserved in both animals and plants and protect their target proteins from denaturation, misfolding and aggregation when subjected to various stresses in organisms^1–7^. HSPs can be classified by molecular weight and sequence similarity into at least five categories, namely HSPp100/ClpB, 90 kDa HSPs, 70 kDa HSPs (HSP70/DnaK), 60 kDa chaperonins (HSP60/GroEL), and sHSPs of 15–42 kDa^8,9^, in which sHSPS are the most abundant and diverse and play important roles in stress tolerance in plants^10–12^. The sHSP family can be further divided into several subfamilies, which target into the cytosol, nucleus, mitochondria, chloroplasts, or peroxisomes in plant cells^3,8,9,13–16^. Such a diversity of plant sHSPs reflect a molecular adaptation to various biotic and abiotic stress conditions. All members of sHSP family are characterized by the presence of a highly conserved alpha-crystallin domain (ACD), which comprises 80–100 amino acid residues and has been thought to function as a central structure element for oligomer (200–800 kDa) formation in native state^9,17–20^. At the upstream of ACD, the N-terminal region appears to be quite flexible among sHSPs and has been thought to be involved in substrate binding and oligomer stability^21–25^.

Under biotic and abiotic stress conditions, a wide range of plant sHSPs have been revealed to act as molecular chaperones *in vitro* and *in vivo* independent of ATP, preventing protein denaturation or aggregation^9,25–28^. In the past decades, the expression of some plant sHSPs are shown to be triggered by heat^29–31^, salt^32^, drought^33^, osmosis^34^, hormones^35^, heavy metal and oxidative stresses^36–39^, or plant developmental signals^40,41^. More evidences have suggested that certain plant sHSPs could enhance the stress tolerance when overexpressing in transgenic plants^42–48^. Despite the identification of diverse functions for various sHSPs in plants, the important roles undoubtedly played by many of these proteins remain to be discovered.

Rice (*Oryza sativa*), an excellent monocot model, is a unique crop feeding nearly half of the population in the world. During its lifecycle, the monocot plant typically has to cope with various stresses, which may greatly reduce rice yield and grain quality. Thus, it should be important to get deep insight into the mechanisms of stress resistance or tolerance in rice. We have previously isolated OsHSP20, a class I cytosolic sHSP from rice (Os03G026700), that interacts with a viral RNA dependent RNA polymerase (RdRp), homodimerizes and also forms granules within the cytoplasm^49^. However, its functions remain unclear. Here we reported the expression pattern of OsHSP20 under various abiotic stresses, its molecular chaperone activity *in vitro* and its role in stress tolerance when overexpressed in *E. coli* (a prokaryote), yeast and rice (eukaryotes). The key domains in the protein crucial for its homodimerization, molecular chaperone activity *in vitro*, and stress tolerance were further determined using a series of truncated mutants.

## Results

### *OsHSP20* was significantly induced by both heat and salt stresses

**Figure 1:**
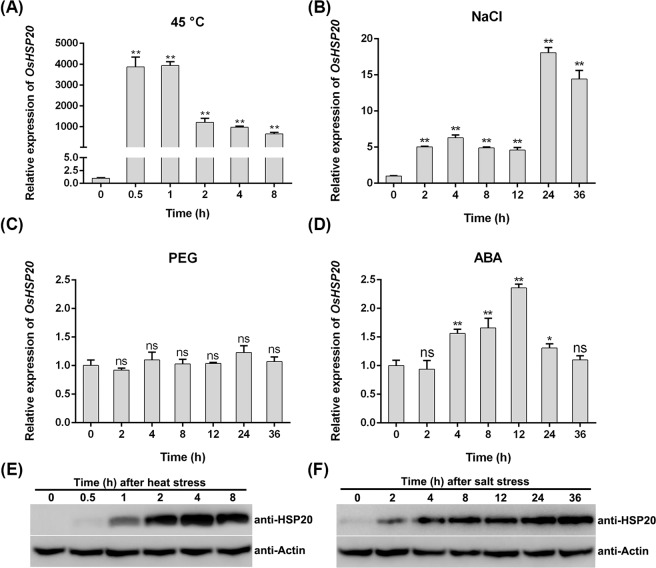
The expression of *OsHSP20* in rice seedlings following various stresses: temperature of 45 °C (heat stress) (**A**), 100 mM NaCl (salinity stress) (**B**), 5% PEG (drought stress) (**C**), or 100 μM ABA (hormone stress) (**D**) analyzed by qRT-PCR (**A**–**D**) and Western blotting (**E,F**), in which the exposure time is 20 s or 150 s for detection of HSP20 protein under heat or salt stress, respectively. Data are mean ± SD from three independent experiments. The asterisks on the top of the columns indicate significant differences from the value at 0 h (ns, not significant; *P  < 0.05; **P < 0.01).

The expression patterns of genes are known to be closely related to their functions and thus could provide a useful clue for exploration of gene function. To reveal the function of *OsHSP20*, its responses to various abiotic stresses, such as heat, salt, drought, and exogenous ABA treatments, were investigated by qRT-PCR. The transcription of *OsHSP20* responded very quickly to heat stress, peaking at approximately 4,000-fold more transcripts than the control within 1 h and then decreasing, although levels were still much higher than the control at 8 h (Fig. 1A). Under salt stress, *OsHSP20* mRNAs were significantly more abundant in the leaves of stressed plants compared to the controls at 2, 4, 8 and 12 h and peaked with values 14–18 fold higher at 24–36 h (Fig. 1B). By contrast, over a 36 h period, the expression of *OsHSP20* showed little or no change (less than 2.3-fold) in response to drought or exogenous ABA treatments (Fig. 1C,D). In Western blotting assays, the OsHSP20 protein was also found to accumulate to much higher level after heat and salt treatments (Fig. 1E,F and Supplementary Figs. S5 and S6).

### Purified recombinant OsHSP20 acts as a molecular chaperone *in vitro*

**Figure 2:**
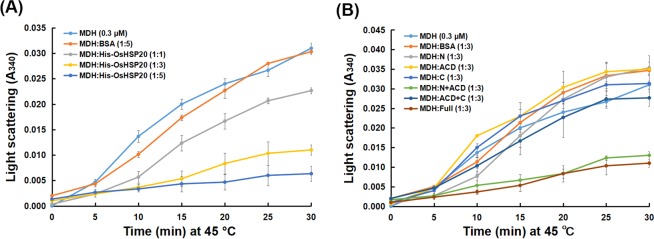
Chaperone activity assay of recombinant full-length (**A**) and truncated (**B**) OsHSP20 proteins. Chaperone activity was measured as the ability to prevent MDH denaturation under thermal-denaturing conditions at 45 °C. BSA was used as negative control. Data are the average of three independent experiments and shown as the mean ± SD (*P* < 0.05). (**A**) MDH (0.3 μM) was incubated in the absence or presence of purified recombinant His-OsHSP20 protein, in which various molar ratios of MDH to His-OsHSP20 (1:1, 1:3 or 1:5) were examined. (**B**) MDH (0.3 μM) was incubated in the absence or presence of truncated OsHSP20 proteins (N, His-OsHSP20N; ACD, His-OsHSP20ACD; C, His-OsHSP20C; N + ACD, His-OsHSP20N + ACD; ACD + C, His-OsHSP20ACD + C) with a molar ratio 1:3 of MDH to recombinant protein, in which purified recombinant His-OsHSP20 protein (Full) was used as positive control.

Some sHSPs have been thought to function as molecular chaperones^16,17^. In order to determine the biochemical characteristics of OsHSP20, a prokaryotic expression vector harboring the full-length coding region of OsHSP20 (pET32a-OsHSP20^Full^) was constructed and expressed in *E. coli*. The expression of recombinant OsHSP20 was induced and validated by SDS-PAGE and western blot assays (Supplementary Fig. S1A,B). The recombinant protein was purified with nickel-sulfate-coupled affinity column and its effect on the aggregation rate of MDH at 45 °C was measured. It was clear that the recombinant OsHSP20 protein markedly inhibited the aggregation of MDH at high temperature (Fig. 2A). It was also notable that different concentration of OsHSP20 had the various effects on the heat-triggered aggregation rate of MDH and the maximum effect (over 80%) was achieved when a molar ratio of MDH to OsHSP20 was equal to 1:5 (Fig. 2A), reflecting the protection in a dose-dependent manner. In contrast, BSA (bovine serum albumin; negative control) at a 1:5 M ratio (MDH:BSA) did not protect MDH from aggregation. This *in vitro* activity suggests that OsHSP20 may function as a molecular chaperonin *in vivo*.

### OsHSP20 enhanced the viability of *E. coli* and yeast cells under thermal and salt stress

**Figure 3:**
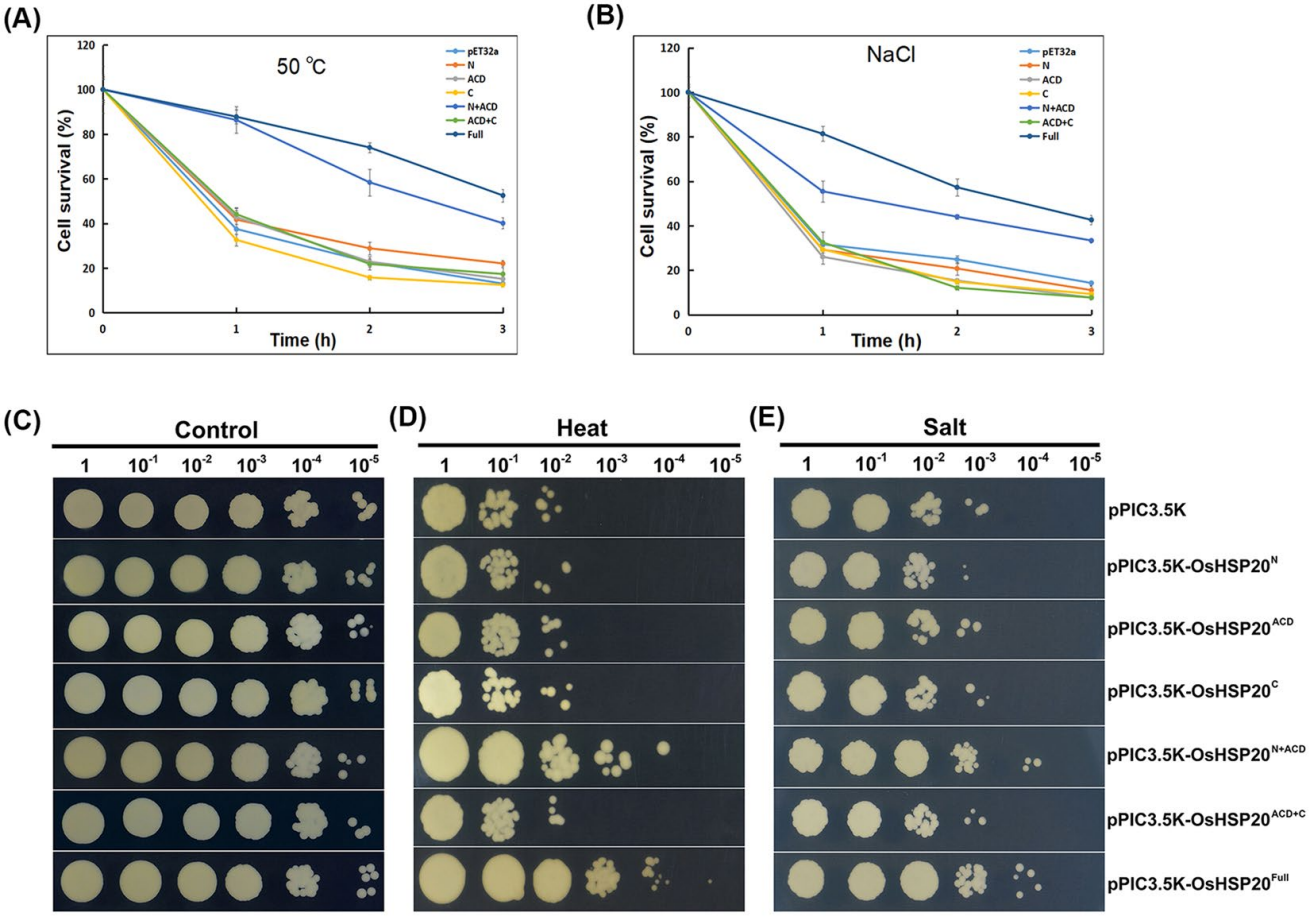
The effects of OsHSP20 overexpression on the growth of *E. coli* BL21 (**A,B**) and *P. pastoris* strain SMD1168 (**C–E**) under thermal and salt stress conditions. (**A,B**) Cell survival of *E. coli* expressing pET32a(+) and transformants (N, pET32a-OsHSP20^N^; ACD, pET32a-OsHSP20^ACD^; C, pET32a-OsHSP20^C^; N + ACD, pET32a-OsHSP20^N+ACD^; ACD + C, pET32a-OsHSP20^ACD+C^; Full, pET32a-OsHSP20^Full^) grown after exposure to high temperature (50 °C) (**A**) or 800 mM NaCl (**B**) for different times (*X*-axis) before shifting to normal conditions for recovery. The data are the average of three independent experiments and shown as the mean ± SD (*P* < 0.05). (**C**–**E**) The SMD1168 strain was transformed with pPIC3.5 K (empty vector), pPIC3.5K-OsHSP20^N^, pPIC3.5K-OsHSP20^ACD^, pPIC3.5K-OsHSP20^C^, pPIC3.5K-OsHSP20^N+ACD^, pPIC3.5K-OsHSP20^ACD+C^ or pPIC3.5K-OsHSP20^Full^. Serially diluted samples (5 μl) were spotted onto plates of YEPD medium after heat (50 °C for 1 h) (**D**) and salt (1.2 M NaCl) (**E**) treatments. The untreated group incubated at 30 °C was used as a control (**C**). Colonies were photographed 3 days after inoculation.

Since above experiments had shown that recombinant OsHSP20 was effectively expressed in *E. coli* BL21(DE3) pLysS transformed with the pET32a-OsHSP20, this overexpression system was used for cell viability assays to investigate the possible functions of OsHSP20 *in vivo*. Under normal conditions, the growth curve of *E. coli* cells with the pET32a-OsHSP20 plasmid appeared to be very similar to that of control cells (data not shown). To investigate the effect of heat stress on survival of recombinant *E. coli* cells, the cultures were subjected to 50 °C for 1–3 h and then shifted to 37 °C for recovery. Cell viability decreased in all cultures subjected to heat shock (50 °C) for 1–3 h, but cells expressing full-length OsHSP20 always survived better than those harboring the empty pET32a(+) vector (Fig. 3A). For example, after a 1 h heat shock approximately 30% of control cells survived compared with 85% of those expressing full-length OsHSP20. The corresponding figures after a 3 h heat shock were respectively 20% compared to nearly 50% (Fig. 3A and Supplementary Fig. S2A). For salt stress tolerance assays, aliquots from IPTG-induced cultures were treated with NaCl and then plated on LB medium. As shown in Fig. 3B, cells expressing the full-length OsHSP20 survived better than the controls at all time-points: after 3 h treatment, over 50% of cells expressing full-length OsHSP20 had survived whereas no more than 20% of control cells had done so (Fig. 3B and Supplementary Fig. S2B). Thus, OsHSP20, a molecular chaperonin, confers thermal and salt tolerance to a representative prokaryote.

To determine the behavior of OsHSP20 as a molecular chaperonin in eukaryotic cells, the coding region of OsHSP20 was sub-cloned into pPIC3.5 K expression vector (designed as pPIC3.5K-OsHSP20^Full^) and transformed into *P. pastoris*, a model yeast cell. The yeast cells transformed with empty pPIC3.5 K vector were used as the control. The effective expression of recombinant OsHSP20 protein in the *P. pastoris* cells was validated by Western blot assays with an anti-HSP20 antibody (Supplementary Fig. S3). Similar viability was observed for test and control recombinant *P. pastoris* cells under normal conditions (Fig. 3C). To evaluate the effect of high temperature on the viability of recombinant *Pichia* cells, 10-fold serial dilutions of cultures were spotted on the YEPD medium plate and then incubated at 50 °C for 1 h before shifted to 30 °C for 2–3 d. It was notable that the yeast cells expressing full-length OsHSP20 grew better than those harboring the pPIC3.5 K vector alone (Fig. 3D). Similarly, the growth of *Pichia* cells expressing full-length OsHSP20 was better than the controls under salt stress (Fig. 3E). It was concluded, therefore, that OsHSP20 also enhances the tolerance to thermal and salt stresses of a eukaryotic unicellular organism.

### The domains required for OsHSP20 homodimerization

**Figure 4:**
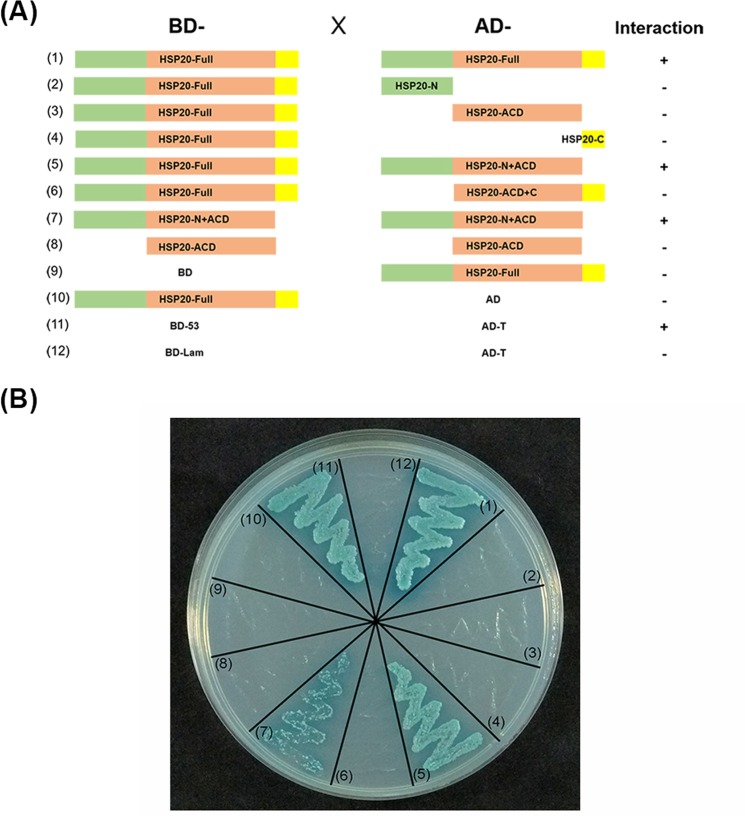
Investigation of OsHSP20 homodimerization in yeast cells. (**A**) Schematic representation of OsHSP20 and the five truncations generated in the study. The full-length OsHSP20 (Full; spanning residues 1 to 158) was divided into three parts: the N-terminal arm (N; spanning residues 1 to 52), the α-crystallin domain (ACD; residues 52 to 143) and the C-terminal extension region (C; residues 143 to 158). Five truncated forms of OsHSP20 were prepared to express N, ACD, C, N + ACD or ACD + C. The presence (+) or absence (−) of homodimerization in YTH assay is shown on the right. (**B**) Interaction of intact OsHSP20 and truncations of OsHSP20 in co-transformed yeast cells grown on SD/-Ade/-His/-Leu/-Trp/X-α-Gal/AbA medium. Yeast colonies expressing BD-53/AD-T were used as the positive control. Yeast co-transformed with BD-Lam/AD-T, BD-OsHSP20^Full^/AD or BD/AD-OsHSP20^Full^ were used as negative controls.

We previously reported that the OsHSP20 protein homodimerizes and forms granules within the cytoplasm^49^. To further determine the key domains of OsHSP20 responsible for homodimerization, we amplified and constructed five truncated mutants of OsHSP20 expressing either the N-terminal region (N), ACD domain, C-terminal region (C), N + ACD or ACD + C (Fig. 4A). These truncated mutants were subcloned into the pGADT7 or pGBKT7 vectors and used for YTH assays. When the C-terminal extension region of OsHSP20 was removed (OsHSP20^N+ACD^), there was no change in homodimerization. The yeast cells co-transformed with the plasmid pairs BD-OsHSP20^Full^/AD-OsHSP20^N+ACD^ and BD-OsHSP20^N+ACD^/AD-OsHSP20^N+ACD^ appeared to grow well and turn blue (Fig. 4B), which were very similar to those of BD-OsHSP20^Full^/AD-OsHSP20^Full^ and the positive control BD-53/AD-T on SD/-Ade/-His/-Leu/-Trp/X-α-Gal/AbA medium (Fig. 4B). There was no visible growth of cells co-transformed with other plasmid combinations showing that the other truncated mutants were unable to homodimerize and therefore that the N-terminal arm plus the ACD domain were required for OsHSP20 dimerization.

### OsHSP20 enhances tolerance to heat and salt stresses in transgenic rice plants

**Figure 5:**
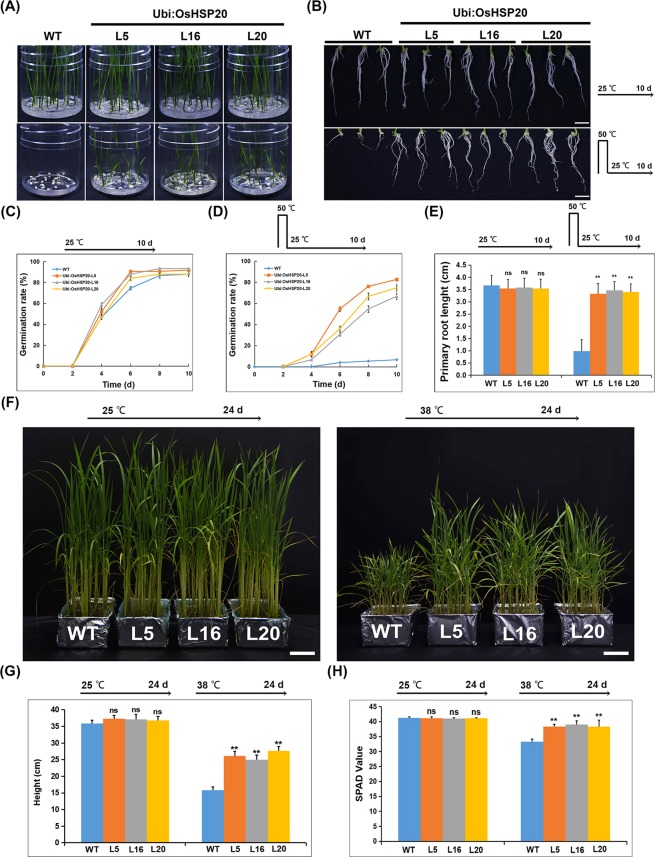
Comparison of seed germination rates (**A**,**C,D**), root lengths (**B**,**E**), plant height (**F,G**), and leaf chlorophyll contents (**H**) from WT and transgenic OsHSP20 rice plants under heat stress. (**A**) Germination rates of untransformed wild-type (WT) and lines overexpressing OsHSP20 (Ubi:OsHSP20-L5, -L16 and -L20) under thermal treatment (50 °C) for 12 h. Photographs were taken after 10 days of recovery at 25 °C. (**B**) Root length phenotype of 10-day-old untransformed wild-type (WT) and lines overexpressing OsHSP20 (Ubi:OsHSP20-L5, -L16 and -L20). Imbibed seeds were treated at 50 °C for 12 h, and photographs were taken after 10 days of recovery at 25 °C. Scale bar is 1 cm. (**C**,**D**) show time course of germination (in days after imbibition) for freshly harvested seeds of WT and the overexpression lines. Seeds were surface-sterilized and plated on 1/2 MS medium under normal growth conditions (25 °C) (**C**) or were first incubated at 50 °C for 12 h and then moved to a growth chamber set at 25 °C (**D**). Germination rate was measured at 2 d intervals after shifting to 25 °C. Data represent the mean ± SD (*P* < 0.05) from three independent experiments. (**E**) Quantitative analysis of primary root lengths of 10-day-old seedlings from WT and each transgenic line. The average ( ± SD) values are from three biological replicates with 30 plants for each line and replicate. Significant differences between WT and transgenic plants are indicated by asterisks (ns, not significant; **P < 0.01). (**F**) Phenotype of transgenic and WT plants grew at 25 °C for 24 days (Left) and 38 °C for 24 days (Right). (**G**) Quantitative analysis of plant height from WT and each transgenic line grew at 25 or 38 °C for 24 days. (**H**) Quantitative analysis of leaf chlorophyll contents from WT and transgenic lines grew at 25 or 38 °C for 24 days.

To investigate the function of OsHSP20 *in vivo* in rice plants, the full-length coding region of OsHSP20 was constructed into a binary vector (p1300-OsHSP20) under the control of the ubiquitin promoter and transformed into rice, generating transgenic plants constitutively overexpressing OsHSP20. Three independent T2 transgenic rice lines (Ubi:OsHSP20-L5, -L16 and -L20) were selected for functional analysis after western blot and qRT-PCR assays showed that both transcripts and proteins of OsHSP20 were accumulated up to higher levels in all of them (Supplementary Figs. S4 and S7). To identify their thermotolerance, the sterilized seeds of three T2 generation transgenic rice lines and the wild-type (WT) control were treated at 50 °C for 12 h on 1/2 MS medium and then shifted to normal growth condition (25 °C) for calculation of their survival rates. When incubated under normal growth conditions (25 °C), both transgenic and WT seeds had almost 90% germinate rates within 10 days (Fig. 5A,C). However, following heat stress (50 °C for 12 h), no more than 10% of the WT seeds had germinated compared to 67–83% in the transgenic lines expressing OsHSP20 (Fig. 5A,D). Thus, OsHSP20 improved the germination of seeds subjected to thermal stress. The primary root lengths of those seedlings that grew were also compared. Under normal growth conditions (25 °C), the OsHSP20 transgenic and WT plants had similar root lengths but growth was inhibited by the heat treatment and the effect was much more severe in the WT than in the transgenic rice plants (Fig. 5B,E). To investigate the effect of heat stress on aerial parts, the transgenic and WT seedlings grew at 25 or 38 °C and their plant height and leaf chlorophyll contents were also analyzed. They almost had similar height and chlorophyll contents when grown at 25 °C, however, the detrimental effect on aerial parts also appeared to be more severe in the WT than in the transgenic seedling when grown at 38 °C (Fig. 5F–H). The results therefore show that transgenic overexpression of OsHSP20 increased thermotolerance in rice during germination and growth development.

**Figure 6:**
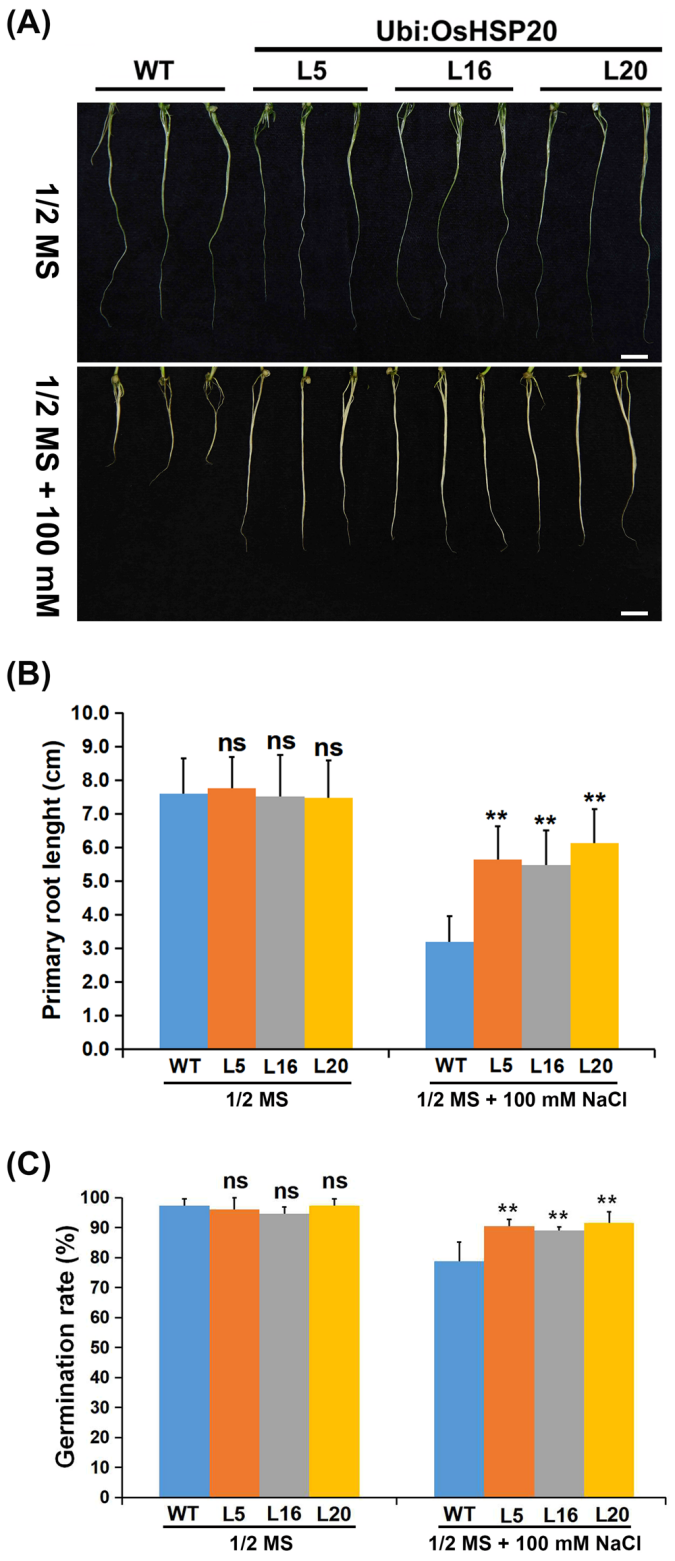
Comparison of root lengths (**A,B**) and seed germination rates (**C**) of transgenic OsHSP20 rice plants under salt stress. (**A**) Root length phenotype of 2-week-old untransformed wild-type (WT) and OsHSP20 overexpression lines (Ubi:OsHSP20-L5, -L16 and -L20). WT and transgenic plants were germinated on 1/2 MS medium plates for 3 days before transfer to a new MS medium plate supplemented with 100 mM NaCl for 2 weeks. Scale bar is 1 cm. (**B**) Quantitative analysis of primary root lengths of 2-week-old seedlings from WT and each transgenic line. The average (±SD) values are from three biological replicates with 50 plants for each line and replicate. Significant differences between WT and transgenic plants are indicated by asterisks (ns, not significant; **P < 0.01). (**C**) Germination rates were measured at 6 d after incubating seeds at 25 °C with or without salt stress. Data represent the mean ± SD (P < 0.05) from three independent experiments.

To investigate the roles of OsHSP20 in defense to salt stress, the germination rate and root elongation was quantified. Under normal growth conditions, the germination rate and root lengths of the transgenic seedlings appeared to be no significantly different from those of WT (Fig. 6). Under salt stress, root growth of WT plants was obviously inhibited (3.2 cm) but this inhibition was significantly alleviated (lengths of 5.5–6.1 cm) by transgenic overexpression of OsHSP20 (Fig. 6A,B). All transgenic lines germinated significantly more than WT under salt stress although treatment with 100 mM NaCl appeared to have mild effect on germination rate (Fig. 6C). Thus, when OsHSP20 was overexpressed it functioned as a molecular chaperonin, conferring heat and salt tolerance to rice.

### The domains required for the chaperone and stress tolerance activity of OsHSP20

To investigate the domains of OsHSP20 required for chaperone activity, the truncated mutants described above were individually constructed into plasmid pET32a(+), a prokaryotic expression vector, and then transformed respectively into *E. coli* for recombinant expression. The purified truncated proteins were then tested for their ability to protect MDH against thermal-induced denaturation. As expected, the full-length protein of OsHSP20 displayed strongest chaperone activity, effectively protecting MDH from thermal-induced aggregation. The N + ACD mutant protein had similar activity to that of the full-length OsHSP20 but none of the other mutant proteins inhibited MDH denaturation (Fig. 2B).

The same truncated mutant constructs were also sub-cloned into pET32a(+) or pPIC3.5 K vectors and transformed into *E. coli* or *P. pastoris* cells, respectively for tests of thermal and salt stress tolerance as described above. In both *E. coli* (Fig. 2B and Supplementary Fig. S2) and *P. pastoris* (Fig. 2D,E and Supplementary Fig. S3) the cells with full-length OsHSP20 and those with the N + ACD mutant survived equally well but there was no evidence of stress tolerance in cells harboring the constructs of N, ACD, C, or ACD + C. Taken together, the results suggested that the N-terminal arm plus the ACD domain was necessary for the homodimerization of OsHSP20 and for its *in vitro* chaperone and stress tolerance activity.

## Discussion

Plant sHSPs are thought to be molecular chaperones that prevent their target proteins or structures from stress-induced aggregation or denaturation, and thus maintain or recover the protein function for plant growth and development under various stresses^3^. Some of sHSP genes, such as *RcHSP17.8* (a *Rosa chinensis* sHSP gene)^44^, *ThHSP18.3* (a *Tamarix hispida* sHSP gene)^50^, and *LimHSP16.45* (a David Lily sHSP gene)^51^, have been reported to be strongly regulated by various types of stresses, including heat, cold, salt, drought, osmotic or oxidative stress^47,48,52,53^. In our experiments, the expression of *OsHSP20* was strongly up-regulated by heat or salt stress but a little or insignificantly affected by ABA treatments or drought stress (Fig. 1), suggesting that it might play different roles in response to the various stresses. The increased transcript abundance of *OsHSP20* in response to heat stress reached a peak in the first hour (Fig. 1A), suggesting that the signal transduction response could be rapid and transient and that such expression pattern of *OsHSP20* might be a kind of mechanism whereby plants rapidly adapt to heat stress. It was also noted that the accumulation of OsHSP20 protein were not always consistent with the transcriptional level of OsHSP20 although they had an increasing tendency under both heat and salt stresses (Fig. 1A,B,E,F). Such differences could be partly caused by the fact that translational process always lags behind the transcription in eukaryotes. The transcriptional level of OsHSP20 appeared to fluctuate greatly with treatment time, however, accumulation of OsHSP20 increased stably, especially in salt treatment, suggesting that its transcription could be regulated by a complexed mechanism *in vivo* and accumulation of stable OsHSP20 proteins might affect its transcription via feedback. By contrast, the signal transduction appeared to be obviously slower in response to salt stress than to heat shock, suggesting a different signal pathway. The endogenous phytohormone abscisic acid (ABA) are well-known to play important roles in response to various stresses^54^. In our experiments, the transcription of *OsHSP20* was a little changed (2.3-fold) by treatment with ABA for 12 h (Fig. 1D), suggesting that *OsHSP20* might be partially involved in the ABA-mediated pathway.

The introduction of plant genes into unicellular organisms is a simple and rapid method for exploration of their putative roles *in vivo*. By this method, some of sHSPs from a few species of plants have been characterized to be specifically involved in defense to certain types of stress tolerances, including *E. coli* expressing CpHSP40 from *Cydia pomonella*^55^, CsHSP17.5 from chestnut (*Castanea sativa*)^43^, or mitochondrial HSP23 from *Medicago sativa*^56^ and *P. pastoris* producing RcHSP17.8 from *Rosa chinensis*^44^ or ThHSP18.3 from *Tamarix hispida*^50^. In this work, we introduced the OsHSP20 coding sequence into both *E. coli* and *P. pastoris* and found that cells overexpressing OsHSP20 were protected to some degree against heat and salt stress. Plant sHSPs have been shown more similar to sHSPs from yeast, a model eukaryotic cell, than to sHSPs of *E. coli*, a prokaryotic cell^57,58^. The effects of OsHSP20 on stress tolerances could be much more balanced by homologous sHSP genes in yeast than in *E. coli* and thus, in this study, the protective effect of OsHSP20 seemed to be more efficient in *E. coli* than that in yeast.

In addition, the OsHSP20 gene was placed under the control of a *Ubi* promoter and transformed by *Agrobacterium*-mediated method into rice plants. Measurements of germination rates and root lengths of rice seedlings showed that the transgenic rice plants were more tolerant of both high temperature (Fig. 5) and salt stress (Fig. 6) than WT control plants. Similar results were also obtained when some sHSPs of several plant species, including *PfHSP21.4* (*Primula*), *ZmHSP16.9* (maize), *PtHSP17.8* (*Populus trichocarpa*) and wheat *sHSP26.8*, were overexpressed in transgenic plants in response to stresses^29,33,34,45,59–62^. However, the detailed molecular mechanism that the OsHSP20 facilitated the germination and root elongation under high temperature and salt stresses remained to be clarified. These transgenic rice lines might provide an excellent plant material for future studies.

Molecular chaperones can bind their target proteins to prevent the denaturation or aggregation of proteins under adverse conditions^3,63^. LimHSP16.45 and another rice sHSP, OsHSP18.2, have been found to act as molecular chaperones to improve cell viability under stress^51,64^. Our results showed that OsHSP20 had the activity of molecular chaperone *in vitro* and enhanced the tolerance to heat and salt stresses, suggesting that the rice sHSP could renature the aggregated mitochondrial malate dehydrogenase (MDH) *in vitro* and that it could protect its target proteins from denaturation induced by stresses in cells. Two other sHSP genes, *AtHSP17.4* and *CsHSP17.2*, have been found to be more actively upregulated in transgenic plants under heat shock^65,66^. We also could not preclude a possibility that transgenic overexpression of *OsHSP20* might induce sHSP synthesis and partially contributed to stress tolerances. Using truncated OsHSP20 mutants, we also showed that the homodimerization of the protein and its *in vitro* biochemical activity and *in vivo* stress tolerance effects all depended on its N-terminal arm plus ACD domains (Figs. 2B and 3). This suggests that its biological activity depends upon its homodimerization. The N-terminal arms of TaHSP16.9, PsHSP18.1 and DgHSP17.2 all have been shown to be crucial for their molecular chaperone activity^23,67^, which is consistent with our results. These results support the view that the N-terminal arm may be essential for the biochemical characteristics of sHSPs, irrespective of subfamily^23^. The N-terminal region has also been thought to be associated with the substrate or target specificity^23^. We therefore suggest that the N-terminal region of OsHSP20 should be important for both its *in vitro* chaperone activity and its stress tolerance *in vivo* by involving homodimerization and interaction with substrates or targets.

## Conclusion

In summary, we have shown that OsHSP20 is induced by heat shock and high salinity stresses. OsHSP20 has molecular chaperone activities *in vitro*. Overexpression of *OsHSP20* in *E. coli*, *P. pastoris* and transgenic rice plants demonstrated that it can enhance heat and salt stress tolerance. Furthermore, the N-terminal region of OsHSP20 was identified to be closely associated not only with its *in vitro* chaperone activity, but also with its *in vivo* stress tolerance.

## Materials and Methods

### Plant materials and abiotic stress treatments

Seedlings of Rice (*Oryza sativa* L. spp. *japonica*. cv. Nipponbare) were grown in a greenhouse at 28 °C with 16 h light/8 h dark cycle and 70% relative humidity (r.h.) for two weeks before treatment. For heat treatment, the seedlings were placed in an incubator at a high temperature (45 °C) or were grown at 28 °C in a culture room as controls. For salinity treatment, the seedlings were watered with 100 mM NaCl or with ddH_2_O as a control. For drought treatment, the roots of seedlings were irrigated with 5% PEG6000 or with ddH_2_O as a control. For the exogenous ABA treatment, the seedlings were sprayed with 100 μM abscisic acid (ABA) or with 0.1% (v/v) ethanol solution (in which the compound was dissolved) as a control. After different times under these stresses, leaf samples were collected, immediately frozen in liquid nitrogen and stored at −80 °C before RNA and protein extraction. Each treatment was represented by three biological replicates, and samples from five plants were collected for each replicate.

### RNA extraction and qRT-PCR

Total RNA was extracted using Trizol reagent (Invitrogen, Carisbad, California, USA) according to the manufacturer’s instructions. The total RNA was first treated with DNase I (TaKaRa Bio, Dalian, China) and then reverse transcribed to cDNA using ReverTra Ace (Toyobo Co., Osaka, Japan) with oligo(dT) primers. 1 μl of 10-fold dilution cDNA from each sample was used for the quantitative analysis with SYBR Green Realtime PCR Master Mix (Toyobo). Data were obtained using the 7900 Real-Time PCR System (Applied Biosystems). The *OsHSP20* gene specific primers Os20RT-F (5′-AAGTTCCTCCGCAGGTTCC-3′) and Os20RT-R (5′-GAGCACGCCGTTCTCCAT-3′) were used in assays. The rice ubiquitin-conjugating enzyme E2 (*UBCE2*) gene (LOC_Os02g42314) was used as an internal control with the primers OsUBC-F (5′-CCGTTTGTAGAGCCATAATTGCA-3′) and OsUBC-R (5′-AGGTTGCCTGAGTCACAGTTAAGTG-3′). The reactions were incubated in a 384-well plate at 95 °C for 3 min and then subjected to 40 cycles of 95 °C for 15 s, 60 °C for 20 s, and 72 °C for 30 s. Each experiment was replicated three times. The relative expression levels were calculated using the 2^−ΔΔCt^ method^68^.

### Protein-protein interaction assays by yeast two-hybrid (YTH) system

The yeast GAL4 binding domain vector pGBKT7 and activation domain vector pGADT7 were used for YTH assays (Clontech, Palo Alto, CA). To construct plasmids for YTH, the coding sequences of the intact OsHSP20 protein and its five truncated mutants OsHSP20^N^ (aa 1–59), OsHSP20^ACD^ (aa 49–145), OsHSP20^C^ (aa 102–158), OsHSP20^N+ACD^ (aa 1–145) and OsHSP20^ACD+C^ (aa 49–158) were amplified separately using primer pairs YOs20-NF/YOs20-CR, YOs20-NF/YOs20-NR, YOs20-ACDF/YOs20-ACDR, YOs20-CF/YOs20-CR, YOs20-NF/YOs20-ACDR and YOs20-ACDF/YOs20-CR (Supplementary Table 1), respectively. The products were then cloned into the pGBKT7 and pGADT7 vectors by *Nde*I/*Bam*HI restriction digestion, creating the bait and prey plasmids, BD- and AD-OsHSP20^Full^, OsHSP20^N^, -OsHSP20^ACD^, -OsHSP20^C^, -OsHSP20^N+ACD^ and -OsHSP20^ACD+C^, respectively.

Yeast transformation was carried out according to the commercial procedures (Matchmaker Gold Yeast Two-Hybrid System; Yeastmaker Yeast Transformation System 2, Clontech). *Saccharomyces cerevisiae* strain Y2HGold was co-transformed with bait and prey plasmids using the small-scale lithium acetate method^69^. Co-transformants were first plated on SD/-Ade/-His/-Leu/-Trp medium, and positive colonies were then tested for α-galactosidase activity on SD/-Ade/-His/-Leu/-Trp/X-α-Gal/AbA medium. The co-transformants with BD-53/AD-T and BD-Lam/AD-T were used as positive and negative controls, respectively. Each YTH assay was performed in at least three replicates.

### Expression and purification of OsHSP20 from *E*. *coli*

The coding sequences of the full-length OsHSP20 protein and its truncated mutants OsHSP20^N^, OsHSP20^ACD^, OsHSP20^C^, OsHSP20^N+ACD^ and OsHSP20^ACD+C^ were amplified separately using primer pairs PEOs20-NF/PEOs20-CR, PEOs20-NF/PEOs20-NR, PEOs20-ACDF/PEOs20-ACDR, PEOs20-CF/PEOs20-CR, PEOs20-NF/PEOs20-ACDR and PEOs20-ACDF/PEOsHSP20-CR (Supplementary Table 1), respectively. The fragments were subsequently ligated into the *Sac*I/*Not*I site of the expression vector pET32a(+) *(Novagen*, Merck, Darmstadt, Germany) and placed in phase with the coding sequence of C-terminal fusion peptide (6 × His). The resulting recombinant plasmids pET32a-OsHSP20^Full^, -OsHSP20^N^, -OsHSP20^ACD^, -OsHSP20^C^, -OsHSP20^N+ACD^, -OsHSP20^ACD+C^ and the empty vector were respectively transformed into *Escherichia coli* BL21(DE3) pLysS cells.

For the expression assay of the recombinant protein, a single clone of transformed *E. coli* was inoculated and cultured at 37 °C overnight in Luria-Bertani medium with ampicillin. The culture was later refreshed to OD_600_ of ~0.5 and then incubated in the presence or absence of isopropyl-β-D-thiogalactopyranoside (IPTG; 1 mM). The *E. coli* cells were harvested and homogenized for lysis. After centrifugation, the lysate was analyzed via sodium dodecyl sulfate polyacrylamide gel electrophoresis (SDS-PAGE)^70^ and coomassie brilliant blue staining.

His-tagged OsHSP20 (His-OsHSP20) and truncated variants were purified by methods of nickel-nitrilotriacetic acid (Ni-NTA, Peptron) resin and subsequent dialysis as described in commercial manual. Their purity was assessed by the Bradford method^71^ using the Bio-Rad protein assay reagent (Bio-Rad, Hercules, CA, USA) and then validated by coomassie brilliant blue staining of the SDS-PAGE gel and western blot analysis.

### Western blot analysis

Western blotting assays were carried out as previously described^49^. Briefly, protein samples (each sample containing 20 μg of protein) were denatured and separated by SDS-PAGE. The electrophoresed proteins were transferred by using a Bio-Rad Mini Protean III transblotting system (Bio-Rad, Hercules, CA, USA) onto nitrocellulose membranes (Millipore, Bedford, MA, USA). The membranes were blocked in TTBS (200 mM Tris, pH 7.0, 1.37 M NaCl, 1% Tween 20, and 3% BSA) for 1 h and then treated with an anti-His monoclonal antibody (1:2,000 dilution; Quanshijin, Beijing, China), an anti-Actin monoclonal antibody (1:2000 dilution; Abbkine, Wuhan, China), or an anti-HSP20 polyclonal antibody (1:2,000 dilution; prepared and stored in our Lab)^72^ as the primary antibody and an anti-rabbit horseradish peroxidase-conjugated secondary antibody (1:5,000 dilution; Kangweishiji, Beijing, China).

### Chaperone activity assays

The chaperone activity of the recombinant His-OsHSP20 and its truncated proteins were evaluated by measuring their ability to prevent the thermal aggregation of malate dehydrogenase (MDH) (EC 1.1.1.37; Sigma) as described previously^73^. Briefly, MDH (0.3 μM) was incubated at 45 °C in a buffer (40 mM HEPES, pH 7.5) alone or with OsHSP20 at a molar ratio of 1:1, 1:3 or 1:5. Absorbance was measured at 340 nm at 5 min intervals for 30 min. For each assay of chaperone activity, BSA was used as the negative control to preclude nonspecific chaperone activity and three independent experiments were performed.

### Survival assay for *E*. *coli* under abiotic stresses

*E. coli* cells were cultivated to the stage (OD_600_ = 1.0) at 37 °C in LB medium and then transferred into fresh LB medium with 100 mg/mL ampicillin and 1 mM IPTG. After expression induced for 2 h, the cultures were shifted to 50 °C for thermotolerance assays. One ml culture samples were respectively taken at 0, 1, 2 and 3 h and then their serial dilutions were plated in triplicate. Cell viability was evaluated by counting average colony-forming units from triplicate plates. For salt stress treatment, aliquots from IPTG-induced cultures were treated with 800 mM NaCl for 1, 2 or 3 h, and then were plated on LB medium. For both heat and salt treatments, *E. coli* cells transformed with empty pET32a(+) vector *(Novagen*, Merck, Darmstadt, Germany) were used as the control. The photographs of colony formation were taken with after culture on plates for 12 h at 37 °C.

### Constitutive expression of OsHSP20 in yeast

*Pichia pastoris* SMD1168 strain was used for constitutive expression of OsHSP20 in yeast as described elsewhere^44^. Fragments of the full-length OsHSP20 and its truncated mutants were digested from the recombinant constructs described above (pET32a-OsHSP20^Full^, -OsHSP20^N^, -OsHSP20^ACD^, -OsHSP20^C^, -OsHSP20^N+ACD^ and -OsHSP20^ACD+C^) via the *Bam*HI/*Not*I sites. These fragments were then introduced into the eukaryotic expression vector pPIC3.5 K (Invitrogen, Carisbad, California, USA) as pPIC3.5K-OsHSP20^Full^, -OsHSP20^N^, -OsHSP20^ACD^, -OsHSP20^C^, -OsHSP20^N+ACD^ and -OsHSP20^ACD+C^, respectively. After being linearized with *Sal*I (Thermo Scientific, Waltham, USA), the recombinant vectors and the empty vector pPIC3.5 K were respectively introduced into the SMD1168 cells with MicroPulser^TM^ electroporator (Bio-Rad, Hercules, CA, USA) and integrated into the genome of SMD1168 cells by homologous recombination. Positive colonies were screened on plates containing yeast extract peptone dextrose (YEPD) medium supplemented with G418 at a concentration of 200 mg/L for 2–3 d of incubation at 30 °C and verified by genomic DNA PCR with the universal primers 5′AOX1 (5′-GACTGGTTCCAATTGACAAGC-3′)/3′AOX1 (5′-GCAAATGGCATTCTGACATCC-3′). SMD1168 cells transformed with empty pPIC3.5 K vector were used as the negative control.

SMD1168 cells were pre-cultivated in buffered glycerol complex medium (BMGY) and inoculated in buffered methanol complex medium (BMMY) as described in commercial manual. When cultivated aerobically to OD_600_ of 1.5, the cultures were used for heat shock treatment at 50 °C for 1 h. At the end of the first hour, 1 ml of culture samples was collected and diluted into a series of 10-fold gradient concentration. Aliquots out of these dilutions were spread in triplicate onto YEPD medium plates for estimating their thermotolerance. For salt treatment, aliquots from the cultures (OD_600_ = 1.5) and dilutions were spread in triplicate on YEPD medium containing 1.2 M NaCl. After incubated in YEPD medium at 30 °C for 2–3 d, survived cells on plates were photographed and evaluated by calculating the colony formation.

### Construction of the plant expression vector and generation of transgenic rice plants

For stable transformation, the encoding region of OsHSP20 was amplified by PCR with the primer pair Trs20-F/Trs20-R (Supplementary Table 1) and then cloned into the pCAMBIA1300 vector^74^ (Sequence Accession Number: AF234296) to produce p1300-OsHSP20. Subsequently, the recombinant vector was transformed into *Agrobacterium tumefaciens* strain EHA105. Rice (cv. Nipponbare) transformation was mediated by agrobacterium as previously described^75^. Briefly, the de-husked seeds were sterilized and cultured on NB medium for callus induction at 26 °C in darkness. The actively growing calli were selected for *Agrobacterium*-mediated transformation. Regenerated transgenic seedlings were transplanted into soil and allowed to grow till maturity inside a greenhouse. To identify the transgenic rice plants, genomic PCR was performed comparing wild type (WT) and transgenic lines with the universal primers p1300-F (5′-TGGCATATGCAGCAGCTATATGTG-3′)/p1300-R (5′-ACTCAGTAGGATTCTG GTGTGTGC-3′). In this study, the T2 generations were further analyzed by qRT-PCR and western blotting before being used in subsequent experiments.

### Analysis of transgenic rice plants under stress conditions

Seeds of three independent transgenic lines (Ubi:OsHSP20-L5, -L16 and -L20) and wild type (WT) were surface sterilized and plated on 1/2 MS medium for stress assays. For heat stress, the plates were treated at 50 °C for 12 h and then transferred to 25 °C for 10 d. For salt stress, the seeds were shifted to 1/2 MS medium with 100 mM NaCl for 14 d after germinated on 1/2 MS medium for 3 d. The germination percentages were scored and the lengths of the roots were measured for estimating the stress tolerances. The chlorophyll contents of leaves were measured with a chlorophyll meter SPAD-502 (Konica-Minolta, Japan) and recorded as SPAD values. All experiments were done at least three repeats and the data for each repeat were measured from more than 30 seeds or seedlings.

## Supplementary information

Supplementary Materials.
