# Supplementary material for: A class I cytosolic HSP20 of rice enhances heat and salt tolerance in different organisms
Source: Sci Rep. 2020 Jan 28;10:1383. doi: 10.1038/s41598-020-58395-8 (PMC6987133; doi:10.1038/s41598-020-58395-8)
Supplement: Supplementary file 1 — Supplementary Materials. [file 41598_2020_58395_MOESM1_ESM.pdf]

# A class I cytosolic HSP20 of rice enhances heat and salt tolerance in different organisms

**Liu-Ming Guo [1, 2, §], Jing Li [1, §], Jing He [1, 2], Han Liu [1, 2], Heng-Mu Zhang [1, 2, *]**

[1] Institute of Virology and Biotechnology, Zhejiang Academy of Agricultural Sciences, Hangzhou 310021, China

[2] College of Chemistry and Life Science, Zhejiang Normal University, Jinhua 321004, China;

**[§]The first two authors contributed equally to this work**

*** Correspondence:** Corresponding authors, E-mail: zhhengmu@tsinghua.org.cn

**Running title:** Chaperone activity of OsHSP20

# Supplementary materials

**Table S1** Primers used in plasmid construction

| Primers | Sequences (5'-3') | Restriction sites[1] |
|---|---|---|
| YOs20-NF | GGAATTCCATATGTCGCTGATCCGCCGC | *Nde*I |
| YOs20-NR | CGCGGATCCCTACTTGAACACGTGCGCCTC | *Bam*HI |
| Y-Os20-ACDF | GGAATTCCATATGTCCTCCGAGACCGCGGCC | *Nde*I |
| Y-Os20-ACDR | CGCGGATCCCTAGACGTCGGGCTTCTTGGC | *Bam*HI |
| YOs20-CF | GGAATTCCATATGCAGGAGGAGAAGACGGAC | *Nde*I |
| Y-Os20-CR | CGCGGATCCCTAGCCGGAGATCTGGATGGA | *Bam*HI |
| PE-Os20-NF | CGAGCTCATGTCGCTGATCCGCCGC | *Sac*I |
| PEOs20-NR | AAGGAAAAAAGCGGCCGCCTACTTGAACACGTGCGCCTC | *Not*I |
| PEOs20-ACDF | CGAGCTCATGTCCTCCGAGACCGCGGCC | *Sac*I |
| PEOs20-ACDR | AAGGAAAAAAGCGGCCGCCTAGACGTCGGGCTTCTTGGC | *Not*I |
| PEOs20-CF | CGAGCTCATGCAGGAGGAGAAGACGGAC | *Sac*I |
| PEOs20-CR | AAGGAAAAAAGCGGCCGCCTAGCCGGAGATCTGGATGGA | *Not*I |
| Trs20-F | CGGAGCTCATGTCGCTGATCCGCCGCAG | *Sac*I |
| Trs20-R | CGCGGATCCTTAGCCGGAGATCTGGATGGAC | *Bam*HI |

[1], underlined

**Figure S1** Characterization of recombinant OsHSP20 in *E. coli*. (A) Heterologous expression of the intact OsHSP20 and its truncated proteins in *E. coli* assessed by SDS-PAGE analysis and Coomassie brilliant blue staining. Line M, protein molecular weight marker; Lane 1: whole cell lysate of non-induced cells containing the empty vector pET32a(+); Lane 2: whole cell lysate of cells containing the empty vector induced with 1 mM IPTG; Lane 3: whole cell lysate of cells containing pET32a-OsHSP20$^{N}$ induced with 1 mM IPTG; Lane 4: whole cell lysate of cells containing pET32a-OsHSP20$^{ACD}$ induced with 1 mM IPTG; Lane 5: whole cell lysate of cells containing pET32a-OsHSP20$^{C}$ induced with 1 mM IPTG; Lane 6: whole cell lysate of cells containing pET32a-OsHSP20$^{N+ACD}$ induced with 1 mM IPTG. Lane 7: whole cell lysate of cells containing pET32a-OsHSP20$^{ACD+C}$ induced with 1 mM IPTG. Lane 8: whole cell lysate of cells containing pET32a-OsHSP20$^{Full}$ induced with 1 mM IPTG. The induced proteins are indicated by black arrows. (B) Western blot analysis of purified recombinant OsHSP20 proteins using anti-His-tag monoclonal antibody. Antibodies were used in dilutions of 1:5000. Lane M, protein molecular weight marker; Lane 1, purified recombinant His-OsHSP20$^{N}$ protein; Lane 2, purified recombinant His-OsHSP20$^{ACD}$ protein; Lane 3, purified recombinant His-OsHSP20$^{C}$ protein; Lane 4, purified recombinant His-OsHSP20$^{N+ACD}$ protein; Lane 5, purified recombinant His-OsHSP20$^{ACD+C}$ protein; Lane 6, purified recombinant His-OsHSP20$^{Full}$ protein. The specific mass (in kDa) of the molecular weight markers is indicated on the left.

**Figure S2** Cell viability of *E. coli* BL21 containing the empty vector pET32a(+) and transformants grown on LB medium after treatment with heat (50 °C) (A) or salt (800 mM NaCl) (B) stresses for different times.

**Figure S3** Western blot analysis with an anti-HSP20 polyclonal antibody demonstrating the presence of the recombinant protein in the *P. pastoris* strain SMD1168 cells. Proteins were extracted from *P. pastoris* cell cultures that were cultivated in buffered methanol complex medium until the optical density at 600 nm ($OD_{600}$) reached 1.4. Antibodies were used in dilutions of 1:5000. M, protein molecular weight marker - the specific mass (in kDa) of the molecular weight marker is indicated on the left; pPIC3.5K, SMD1168 cells carrying empty vector; N, SMD1168 cells carrying vector pPIC3.5K-OsHSP20$^{N}$; ACD, SMD1168 cells carrying vector pPIC3.5K-OsHSP20$^{ACD}$; C, SMD1168 cells carrying vector pPIC3.5K-OsHSP20$^{C}$; N+ACD, SMD1168 cells carrying vector pPIC3.5K-OsHSP20$^{N+ACD}$; ACD+C, SMD1168 cells carrying vector pPIC3.5K-OsHSP20$^{ACD+C}$; Full, SMD1168 cells carrying vector pPIC3.5K-OsHSP20$^{Full}$.

**Figure S4** qRT-PCR and western blot analysis of transgenic OsHSP20 rice lines. (A) qRT-PCR analysis. Total RNA was extracted from leaves of 4-week-old WT and T2 generation transgenic OsHSP20 rice. The *OsUBCE2* (LOC_Os02g42314) gene was used as an internal control. (B) Western blot analysis using an anti-HSP20 polyclonal antibody. Proteins were extracted from leaves of 4-week-old WT and T2 generation transgenic OsHSP20 rice. Western blot analysis using anti-Actin monoclonal antibody was used as an internal control. Antibodies were used in dilutions of 1:5000. WT, wild-type; Ubi:OsHSP20-L5, -L16 and -L20, three transgenic rice lines.

**Figure S5** Integral blots of Figure 1E for detection of OsHSP20 in rice seedlings following heat stress (45 °C), in which the exposure time is 20 s for OsHSP20 protein (A) and 30 s for Actin (B).

**Figure S6** Integral blots of Figure 1F for detection of OsHSP20 in rice seedlings following salinity stress (50 mM NaCl)， in which the exposure time is 150 s for OsHSP20 protein (A) and 30 s for Actin (B).

**Figure S7** Integral blots of Figure S4B for detection of OsHSP20 in transgenic rice plants, in which the exposure time is 30 s for both OsHSP20 protein (A) and Actin (B).

Figure S1

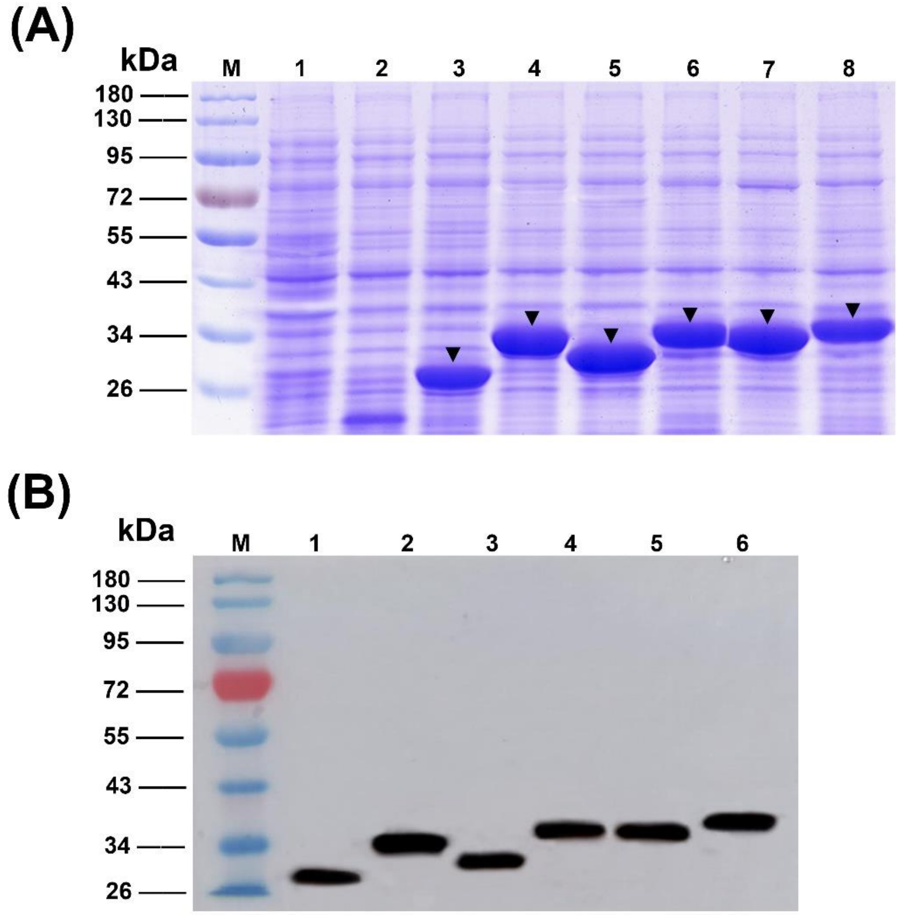
(A)
kDa
M 1 2 3 4 5 6 7 8
180
130
95
72
55
43
34
26
(B)
kDa
M 1 2 3 4 5 6
180
130
95
72
55
43
34
26

**Figure S2**

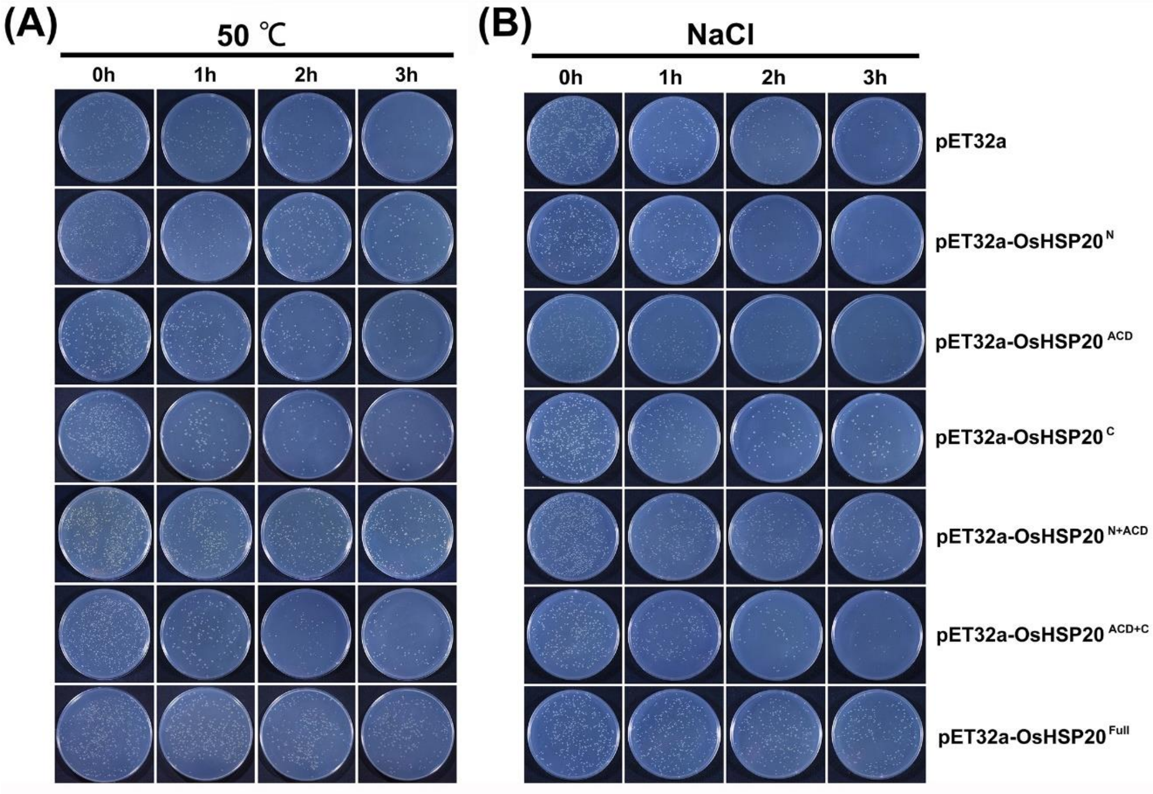
(A)
50 ℃
0h
1h
2h
3h
(B)
NaCl
0h
1h
2h
3h
pET32a
pET32a-OsHSP20 N
pET32a-OsHSP20 ACD
pET32a-OsHSP20 C
pET32a-OsHSP20 N+ACD
pET32a-OsHSP20 ACD+C
pET32a-OsHSP20 Full

**Figure S3**

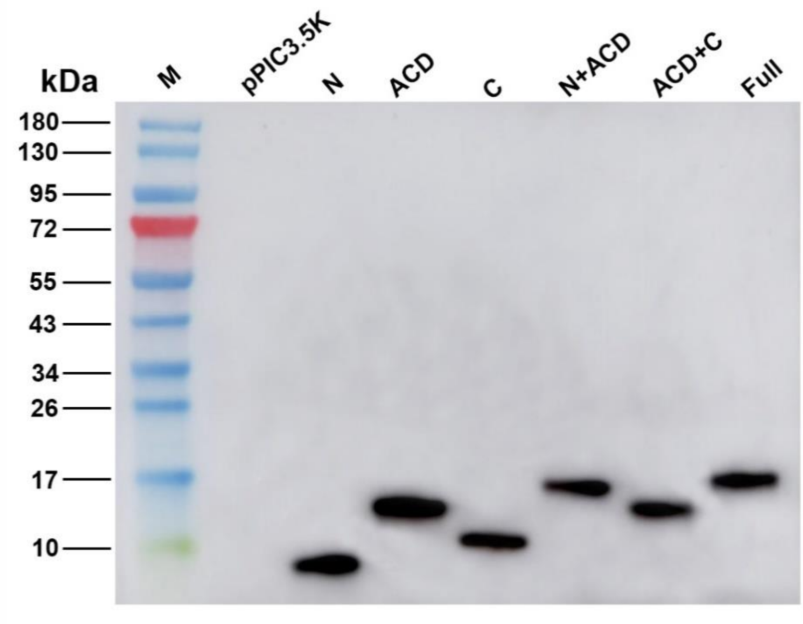
kDa
M
pPIC3.5K
N
ACD
C
N+ACD
ACD+C
Full
180
130
95
72
55
43
34
26
17
10

**Figure S4**

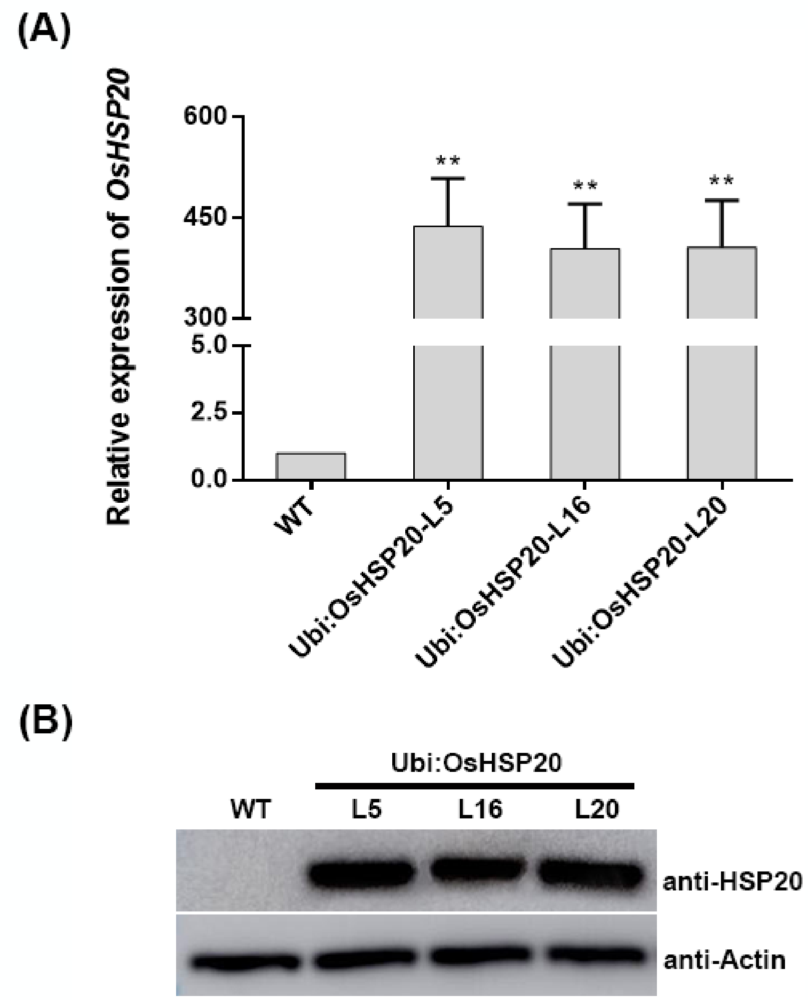
(A)
Relative expression of OsHSP20
600
450
300
5.0
2.5
0.0
**
**
**
WT
Ubi:OsHSP20-L5
Ubi:OsHSP20-L16
Ubi:OsHSP20-L20
(B)
Ubi:OsHSP20
WT
L5
L16
L20
anti-HSP20
anti-Actin

**Figure S5**

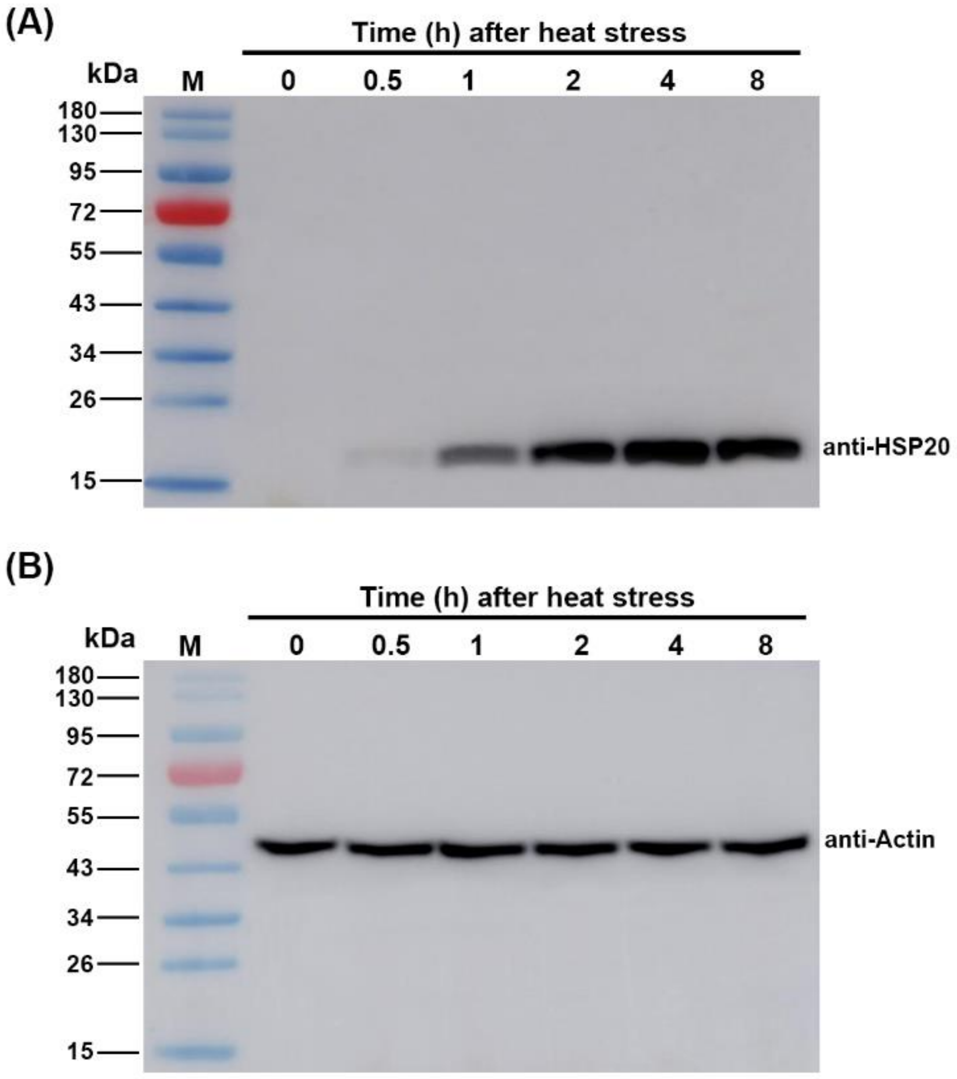
(A)
Time (h) after heat stress
kDa
M
0
0.5
1
2
4
8
180
130
95
72
55
43
34
26
15
anti-HSP20
(B)
Time (h) after heat stress
kDa
M
0
0.5
1
2
4
8
180
130
95
72
55
43
34
26
15
anti-Actin

**Figure S6**

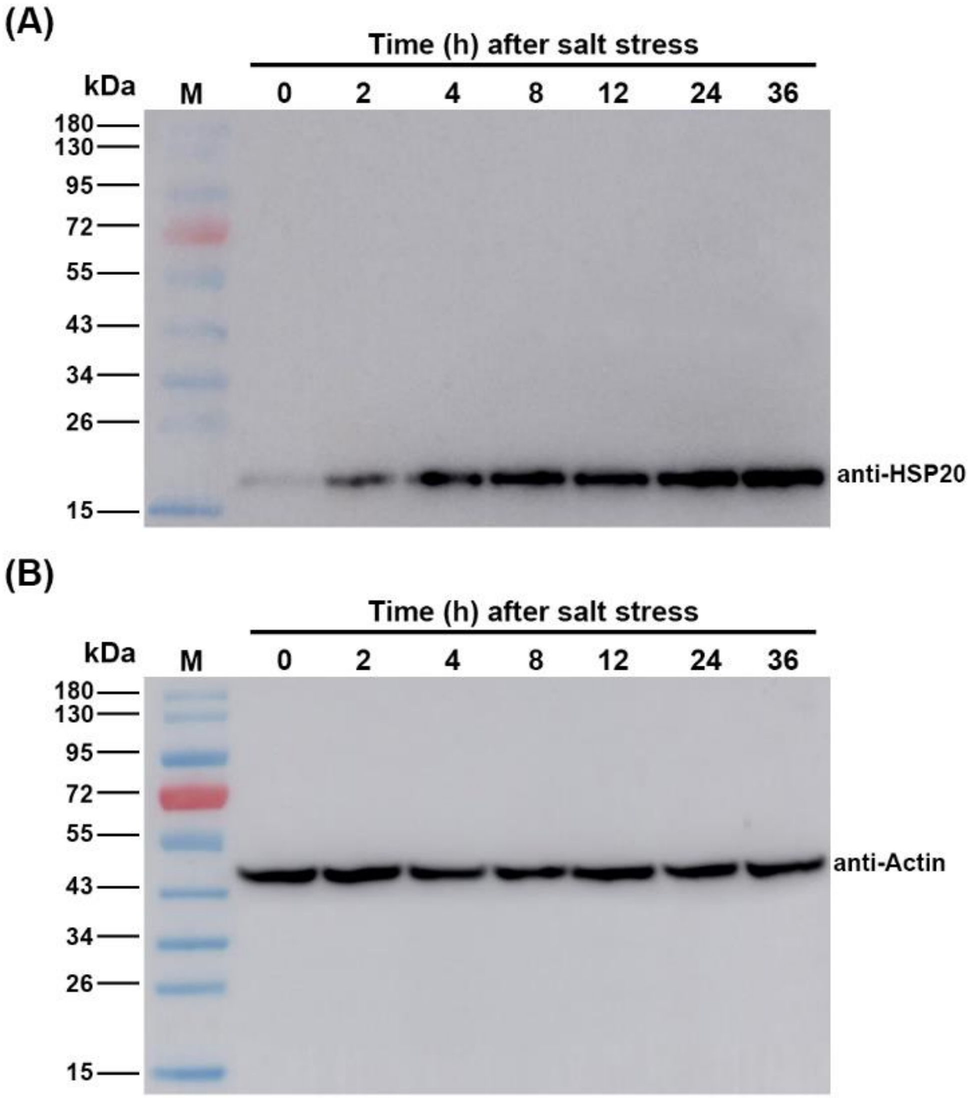
(A)
Time (h) after salt stress
kDa
M
0
2
4
8
12
24
36
180
130
95
72
55
43
34
26
15
anti-HSP20
(B)
Time (h) after salt stress
kDa
M
0
2
4
8
12
24
36
180
130
95
72
55
43
34
26
15
anti-Actin

**Figure S7**

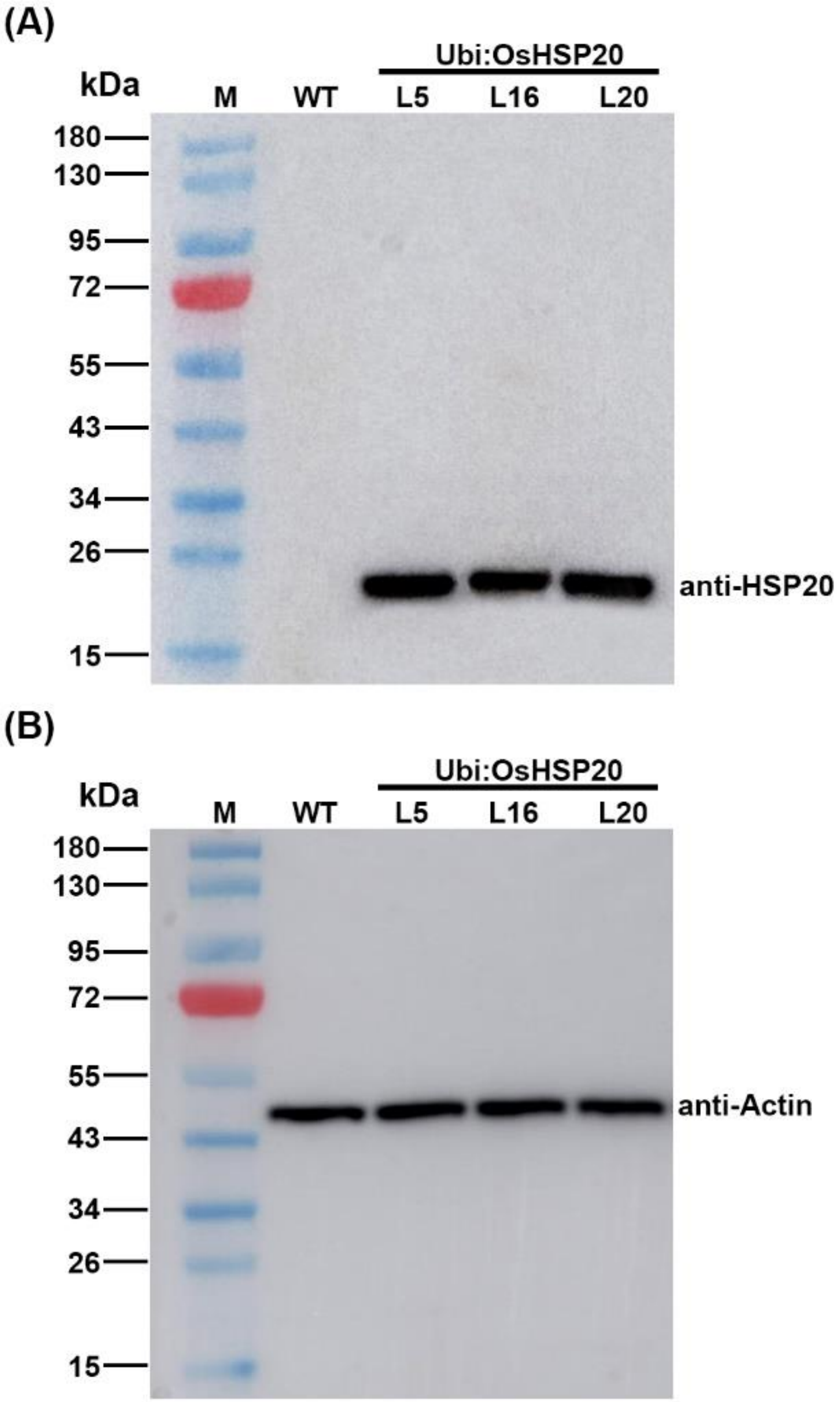
(A)
Ubi:OsHSP20
kDa
M
WT
L5
L16
L20
180
130
95
72
55
43
34
26
15
anti-HSP20
(B)
Ubi:OsHSP20
kDa
M
WT
L5
L16
L20
180
130
95
72
55
43
34
26
15
anti-Actin
